# Identification and validation of m6A-GPI signatures as a novel prognostic model for colorectal cancer

**DOI:** 10.3389/fonc.2023.1145753

**Published:** 2023-06-23

**Authors:** Bin Ma, Simeng Bao, Yongmin Li

**Affiliations:** ^1^ Department of Colorectal Surgery, Cancer Hospital of China Medical University, Cancer Hospital of Dalian University of Technology, Liaoning Cancer Hospital and Institute, Shenyang, China; ^2^ The Liaoning Provincial Key Laboratory of Interdisciplinary Research on Gastrointestinal Tumor Combining Medicine with Engineering, Shenyang, China; ^3^ Central Laboratory, Cancer Hospital of China Medical University, Cancer Hospital of Dalian University of Technology, Liaoning Cancer Hospital and Institute, Shenyang, China

**Keywords:** colorectal cancer, N6-methyladenosine, prognostic signature, immune infiltration, CIITA

## Abstract

In order to develop an N6-methyladenosine-related gene prognostic index (m6A-GPI) that can predict the prognosis in colorectal cancer (CRC), we obtained m6A-related differentially expressed genes (DEGs) based on The Cancer Genome Atlas (TCGA) and m6Avar database, seven genes were screened by weighted gene co-expression network analysis (WGCNA) and least absolute shrinkage and selection operator (LASSO) analysis. Then, m6A-GPI was constructed based on the risk score. Survival analysis indicated that patients in the lower m6A-GPI group have more prolonged disease-free survival (DFS), and different clinical characteristic groups (tumor site and stage) also showed differential risk scores. In the analysis of the molecular characteristics, the risk score is positively associated with homologous recombination defects (HRD), copy number alterations (CNA), and the mRNA expression-based stemness index (mRNAsi). In addition, m6A-GPI also plays an essential role in tumor immune cell infiltration. The immune cell infiltration in the low m6A-GPI group is significantly higher in CRC. Moreover, we found that CIITA, one of the genes in m6A-GPI was up-regulated in CRC tissues based on real-time RT-PCR and Western blot. m6A-GPI is a promising prognostic biomarker that can be used to distinguish the prognosis of CRC patients in CRC.

## 1 Introduction

Colorectal cancer (CRC) has risen rapidly in recent years (1–3), and the treatment of CRC is mainly based on surgery, targeted therapy, neoadjuvant chemoradiotherapy, neoadjuvant radiotherapy and adjuvant chemotherapy. Unfortunately, current treatments for CRC remain limited (4–6). Precision oncology enables the administration of therapies to specific subsets of patients who exhibit the most favorable responses based on their characteristics. Prognostic models based on prognostic biomarkers have been employed for clinical decision-making and prognostication of therapeutic response (7). Moreover, some studies also have proposed the utilization of intelligent technologies and prognostic biomarkers to provide essential guidance of precision therapy (8, 9). Therefore, we need to identify those high-risk CRC patients with poor prognosis, and further clarify the relevant mechanisms, so that individualized treatment can be implemented as soon as possible.

The tumor immune microenvironment (TIME) has an important impact on tumor prognosis and therapeutic effect (10, 11). As the most abundant internal modification of eukaryotic mRNA and non‐coding RNA, N6-methyladenosine (m6A) modification is not only associated with tumor growth, proliferation, and metastasis but also affects the process of immune cell recruitment and metabolic regulation of the TIME (12, 13), which will seriously affect the prognosis in CRC. The modification of m6A is performed by m6A writers, erasers and reader. Among these, METTL3 and METTL14 make up the majority of m6A methyltransferases (m6A writers). YTHDF1 is a m6A reader protein that promotes the translation of m6A-modified mRNA (14, 15). Some studies have shown that blocking METTL3 can enhance the chemotherapeutic response and reduce stem cell frequency and tumor size both in vitro and in vivo (16). Han et al. found that mice with blockade of YTHDF1 show an elevated antigen-specific CD8^+^ T cell antitumor response compared with wild-type mice, and the therapeutic efficacy of programmed cell death-ligand 1 (PD-L1) checkpoint blockade is enhanced in YTHDF1^-/-^ mice, which implies that YTHDF1 can serve as a potential therapeutic target in tumors (17). In another study, the consumption of METTL3 or METTL14 in CT26 tumor mice with anti-PD-1 therapy significantly slowed tumor proliferation and prolonged the survival rate (18). These results suggest that m6A modification may serve as a target affecting immune infiltration and survival time in CRC patients.

Despite this, there is no reliable tool for predicting the prognosis in CRC, and effective indicators are urgently needed. In this study, we developed a prognostic biomarker that can predict the prognosis and the immune infiltration in CRC patients. We focused on m6A-related genes, and seven genes were screened. Then, we established an m6A-related gene prognostic index (m6A-GPI) based on the risk score. We conducted a series of stratification analysis and revealed the molecular and immune cell infiltration characteristics in the m6A-GPI subgroups. In addition, m6A-GPI was an independent predictor for CRC, and we constructed a nomogram including m6A-GPI to help clinicians accurately predict the prognosis of CRC patients. Moreover, we found that CIITA, one of the genes in m6A-GPI was up-regulated in CRC tissues based on real-time RT-PCR and Western blotting. These results showed that m6A-GPI is a reliable biomarker for predicting the prognosis of CRC.

## 2 Methods

### 2.1 Datasets acquisition

We downloaded the RNA-seq data and clinical features of CRC from The Cancer Genome Atlas (TCGA) (https://portal.gdc.cancer.gov) database, which contained 616 tumor tissue samples and 51 paracancerous tissue samples. Mutation data were downloaded using the “TCGAbiolinks” packages in R language, and the independent validation datasets (GSE17538, 200 samples) were obtained from Gene Expression Omnibus (GEO) (https://www.ncbi.nlm.nih.gov/geo/).

### 2.2 Identification of m6A-related genes

Then, pertinent references were searched and 21 m6A-related genes were screened, we identified 6,797 m6A-related genes in colorectal cancer from the m6Avar database (http://rmvar.renlab.org/). The differential expression analysis was performed in the "limma" R package, and differentially expressed genes (DEGs) were obtained in this process (adj. *P*< 0.05, log2FC > 0.585 or< 0.67). Consensus clustering, Gene Ontology (GO) and Kyoto Encyclopedia of Genes and Genomes (KEGG) analysis were then performed in Metascape (https://metascape.org/) after consideration in the context of the m6A-related genes obtained from TCGA and m6Avar.

### 2.3 WGCNA analysis

We conducted WGCNA analysis to identify hub genes. First, the similarity matrix was transformed into an adjacency matrix and then into a topological overlap matrix (TOM); TOM distances were used to cluster genes into WGCNA modules, and modules were determined by the dynamic pruning tree with a minimum of 30 genes per module.

### 2.4 Construction and validation of m6A-GPI

In order to identify prognostic genes, using the R package “survival” to perform univariate Cox regression analysis. Next, we implemented LASSO Cox regression to construct m6A-GPI that can predict the disease-free survival (DFS) of CRC patients, m6A-GPI was calculated based on the coefficient of genes, and the formula is:


$$risk\;score=\sum_{i=\;1}^{n}Coef_{i}*x_{i}$$

where $Coef_{i}$ is the coefficient, and the $x_{i}$ is the FPKM value of the m6A-related genes. We constructed Kaplan–Meier (K-M) survival curves of two subgroups and analyzed the 1-, 3-, and 5-year DFS rates of the cases. Validation datasets were downloaded from the GEO database, and we combined TCGA clinical information and explored the stability of m6A-GPI with different clinical characteristics.

### 2.5 Comprehensive analysis of molecular and immune characteristics in different m6A-GPI subgroups

DNA changes are the basic factor in the development of cancer and play an important role in promoting the progress of cancer (19), and tumor immune escape mechanisms indicate that malignant tumors are capable of evading the immune response. To explore the immunogenicity of CRC, we analyzed the effect of m6A-GPI on mutation load, homologous recombination defects (HRD), neoantigen loads, copy number alterations (CNA) and the mRNA expression-based stemness index (mRNAsi). We obtained immune characteristics from the Genomic Data Commons (GDC) data portal (https://gdc.cancer.gov/about-data/publications/panimmune), and the HRD score comes from PMID: 29617664. Furthermore, we analyzed the somatic mutation difference between the low- and high-risk groups by the R package “maftools.”

The CIBERSORT (https://cibersort.stanford.edu/) is a novel algorithm and it can evaluate gene expression data from RNA sequences and assess the immune cell compositions of complex tissues (20, 21). CIBERSORT can be used to calculate the content of 22 kinds of human immune cell phenotypes and the sum of all estimates of immune cell type fractions yields one. We compared the relative proportions of 22 immune cells between the two subgroups and presented the results in a landscape map.

In the tumor microenvironment, non-tumor components are divided into two types that are valuable for tumor diagnosis and prognostic evaluation, immune cells and stromal cells (22). To determine the impact of immune cell infiltration (such as T cells, Tregs, NK cells and macrophages) on the treatment of ICIs, we calculated the immune score, matrix score, and tumor purity in each CRC sample based on the ESTIMATE algorithm.

### 2.6 Independent prognostic factor and nomogram

To verify whether m6A-GPI can serve as an independent prognostic factor in CRC, we conducted univariate and multivariate Cox regression analysis. In order to provide doctors with a quantitative method for predicting the prognosis of patients with CRC, we constructed a nomogram using the risk status, age, cancer type, sex, cancer stage, and cancer site, then established calibration plots of DFS at 1-, 3-, and 5-year in the TCGA cohorts.

### 2.7 CRC tissue samples

Colorectal samples and their adjacent normal tissues were collected from Liaoning Cancer Hospital and Institute (Shenyang, China), and the colorectal samples showed a confirmed histological diagnosis of CRC. The study was approved by the institutional ethics committee, and individual consent forms were signed by each patient.

### 2.8 Quantitative real-time RT-PCR

Total RNA was extracted from colorectal tumors and their adjacent normal tissues of 16 patients by using Trizol reagent (Life Technologies, Carlsbad, CA) following the manufacturer recommendations. Concentration of RNA was quantified by Nanodrop 2000 (Thermo, Wilmington, DE). Reverse-transcription to cDNA (50 ng per sample) was using with iScript cDNA Supermix (TaKaRa, Dalian, China). Quantitative RT-PCR was performed using a reaction mixture containing SYBR mix (TaKaRa, Dalian, China), and real-time fluorescence was detected by Quant Studio 6 Flex (ABI, Foster City, CA). The primers were designed and synthesized by Life Technologies. The sequences of the primer pairs were as follows, *GBP2*: forward 5′-CTATCTGCAATTACGCAGCCT-3′, reverse 5′-TGTTCTGGCTTCTTGGGATGA-3′, *CXCL10*: forward 5′-GTGGCATTCAAGGAGTACCTC-3′, reverse 5′-TGATGGCCTTCGATTCTGGATT-3′, *CXCL13*: forward 5′-GCTTGAGGTGTAGATGTGTCC-3′, reverse 5′-CCCACGGGGCAAGATTTGAA-3′, *FASLG*: forward 5′- TGCCTTGGTAGGATTGGGC-3′, reverse 5′-GCTGGTAGACTCTCGGAGTTC-3′, *CIITA*: forward 5′-CCTGGAGCTTCTTAACAGCGA-3′, reverse 5′-TGTGTCGGGTTCTGAGTAGAG-3′, *IL12RB1*: forward 5′-TAGGGACCTGAGATGCTATCG-3′, reverse 5′-CCCGGAGCTAAGGCAACAC-3′, *CXCR6*: forward 5′-GACTATGGGTTCAGCAGTTTCA-3′, reverse 5′-GGCTCTGCAACTTATGGTAGAAG-3′, *GAPGH*: forward 5′-TCCCATCACCATCTTCCA-3′, reverse 5′-ACTCACGCCACAGTTTCC-3′.

### 2.9 Western blot analysis

CRC samples and their adjacent normal tissues of 7 patients were lysed according to the kit (PC101-PC104, Epizyme Biomedical Technology, Shanghai, China). Total protein concentration was quantified using a BCA protein assay kit (DQ111-01, TransGen Biotech Co., Ltd., Beijing, China). 50 μg of protein was separated by SDS–PAGE and was electrophoretically transferred to polyvinylidene difluoride (PVDF) membranes. The membranes were blocked with 3% BSA in Tris-buffered saline (TBS) containing 0.05% Tween-20 for 1 h. After blocking, the membranes were incubated with corresponding primary antibodies overnight at 4°C. The primary antibody for CIITA (#55099-1-AP; 1:1000) was purchased from Proteintech Group, Inc. (Wuhan, China). Membranes were washed by 1× TBST, followed by incubation with anti-Rabbit IgG-HRP (ZB-2301, Zhong Shan-Golden Bridge Biological Technology Co., Beijing, China) for 1 h. Immunoreactive bands were visualized by using Tanon 5500 (Tanon, Shanghai, China). Equal loading of proteins was verified by GAPDH (#60004-1-Ig; 1:3000, Proteintech Group, Inc., Wuhan, China).

### 2.10 Statistical analysis

SPSS 24.0 (IBM Corporation, Armonk, United States) and R programming language (version 4.0.2) were used to perform the statistical analysis. Kaplan–Meier curves and the log-rank test were used to compare the DFS between various subgroups. The prognostic ability of the predictors for 1-, 3-, and 5-year DFS was evaluated by receiver operating characteristic (ROC) curves and the area under the curve (AUC) values. Univariate and multivariate Cox regression analysis were utilized to evaluate the independent prognostic value of the model. A two-sided *P*< 0.05 was considered significant.

## 3 Results

### 3.1 Identification of m6A-related genes

**Figure 1:**
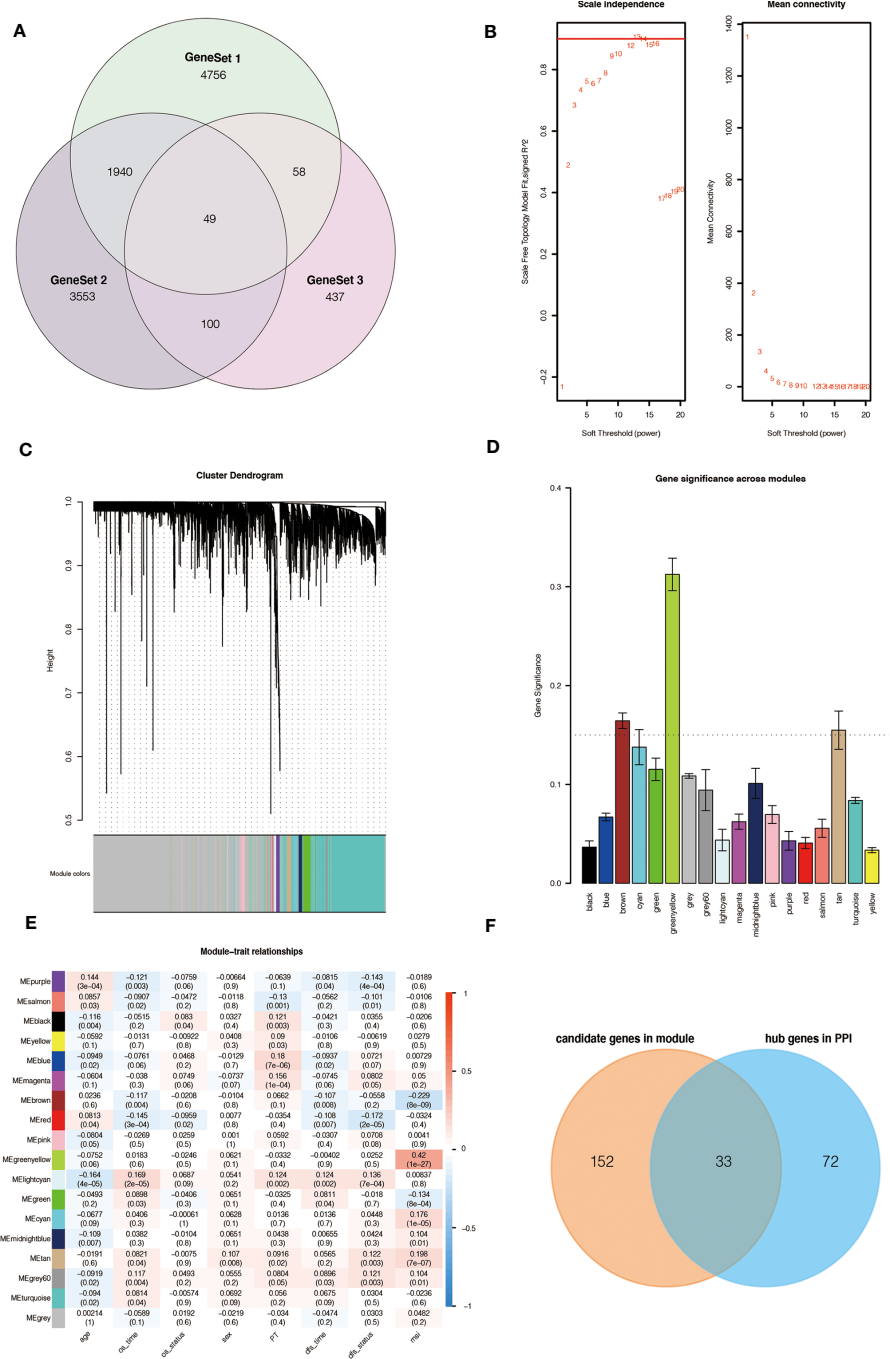
Source of m6A-related genes and weighted correlation network analysis. **(A)** Venn diagram of the three gene sets. **(B)** Identification of soft-thresholding power for the scale-free network. **(C)** Clustering dendrogram and merging of co-expression modules. **(D)** Gene significance of each module. **(E)** The correlation heatmap of mRNA modules and clinical traits is related to color changes. Red represents positive correlation, and blue represents negative correlation. **(F)** Venn diagram of the candidate genes in the green-yellow and brown modules and the hub genes in the PPI network.

Based on our workflow (**Supplementary Figure 1**), we obtained 6,803 m6A-related and referred to as GeneSet 1. These genes intersected with 5,220 DEGs from the TCGA database, and 1,291 differentially expressed m6A-related genes were screened for GO and KEGG analysis. In the KEGG and GO analysis, we extracted a total of 5,642 genes from the significantly enriched pathways, which were referred to as GeneSet 2. In addition, we also clustered 1,291 differentially expressed m6A-related genes, and finally obtained two sets of samples, and screened out 644 DEGs between them, which were referred to as GeneSet 3. The three gene sets contained a total of 10,893 genes (**Figure 1A**). The screening process is shown in **Supplementary Figure 2**.

### 3.2 33 key genes were identified by WGCNA analysis

To obtain key genes related to m6A modification, we performed WGCNA on 10,893 genes and finally obtained 18 modules (**Figures 1B, C**). Then, the Gene Significance (GS) value of each module was calculated, with a larger GS indicating that the module was more related to the phenotypic characteristics of the sample (**Figure 1D**). We calculated the Pearson correlation coefficient between each module and the phenotypic characteristics of the sample (**Figure 1E**).

According to the results, we identified two key modules, green-yellow and brown, and selected the genes in these two modules for subsequent analysis. We constructed a protein-protein interaction (PPI) network based on two modules, and 105 hub genes were screened in the network (**Supplementary Figure 3A**). At the same time, 185 hub genes were screened in these two modules according to the thresholds of MM > 0.6 and GS > 0.2 (**Supplementary Figure 3B, C**). The intersection contains 33 genes, which are considered to be the key genes related to m6A (**Figure 1F**).

### 3.3 Construction of the m6A-GPI in the TCGA dataset

**Figure 2:**
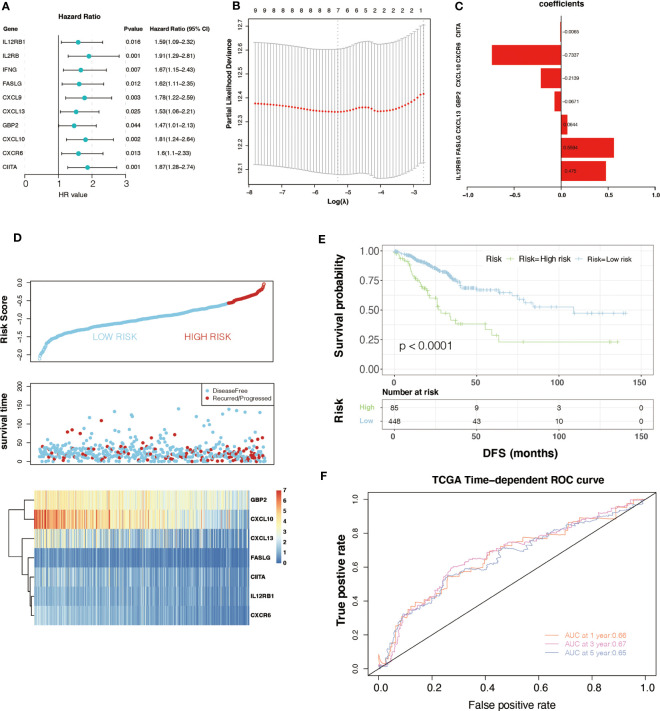
Univariate Cox regression analysis and the prognostic model. **(A)** Univariate Cox regression analysis indicated that ten genes play a critical role in the prognosis of colorectal cancer. **(B, C)** The calculation of minimum criteria and the coefficients. **(D)** The distribution of risk score and the status of colorectal cancer patients and the heatmap of hub genes. **(E)** Kaplan–Meier curves showed that the patients in the high-risk group had worse DFS in the TCGA dataset. **(F)** ROC curves of m6A-GPI for predicting the 1-, 3-, and 5-year survival in the TCGA dataset.

Univariate Cox analysis was performed on 33 key genes, and the results showed that 10 genes (IL12RB1, IL2RB, IFNG, FASLG, CXCL9, CXCL13, GBP2, CXCL10, CXCR6, and CIITA) had a significant relationship with the prognosis of CRC (*P*< 0.05). The forest plot is presented (**Figure 2A**). To further determine the genes used to construct m6A-GPI, LASSO analysis was performed to identify the 7 most important genes and their coefficients (IL12RB1, FASLG, CXCL13, GBP2, CXCL10, CXCR6, and CIITA) (**Figure 2B, C**), we utilized m6A-GPI to determine the patient’s risk score and divided all patients into a high-risk group and a low-risk group based on the median risk score (**Figure 2D**).

Compared with patients with high-risk scores, lower risk scores represent better DFS and a relatively longer survival time in the K-M curves (*P*< 0.05) (**Figure 2E**). At the same time, a ROC curve was used to test the accuracy of m6A-GPI in predicting patient survival. The AUC of the 1-, 3-, and 5-year DFS rates reach 0.66, 0.67 and 0.65, respectively (**Figure 2F**), which indicated that m6A-GPI has the potential to predict the DFS of patients in the TCGA cohort.

### 3.4 Validation of the m6A-GPI

**Figure 3:**
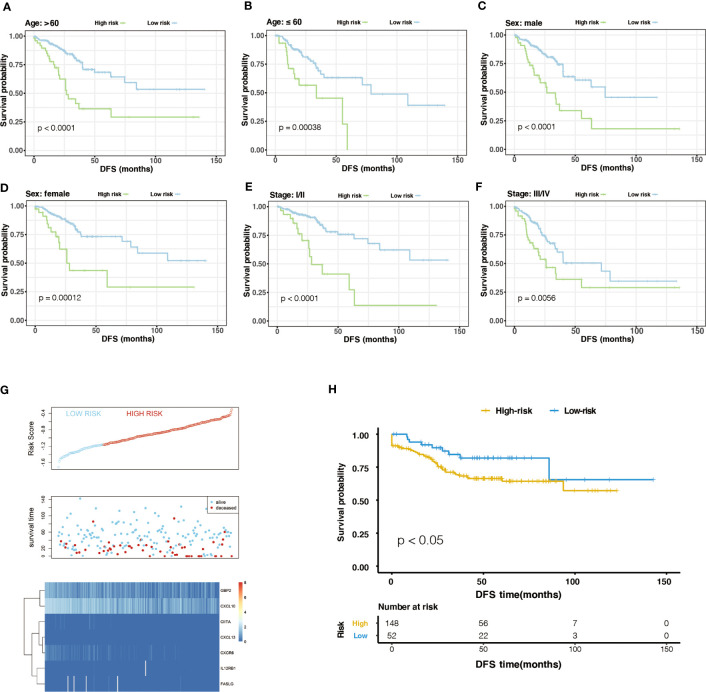
The stratification analysis and validation of the GEO dataset. **(A-F)** The stability of m6A-GPI in stratified samples (divided by age, sex, and stage). **(G)** The distribution of risk score and the status of colorectal cancer patients and the heatmap of hub genes in the GEO dataset. **(H)** Kaplan–Meier curves showed that the patients of the high-risk group had worse DFS in the GEO dataset.

Generally, the pathological stage is of great significance to the prognosis of CRC (23), but other factors such as age and gender can also affect the prognosis. Therefore, we tested the stability of m6A-GPI in different clinical characteristics. In the stratified samples based on GEO dataset, the results showed that the high- and low-risk groups still had significant survival differences after distinguishing age, sex, and stage (*P*< 0.05) (**Figure 3A–F**), which indicates that the m6A-GPI has good stability in stratified samples.

The external dataset was obtained from the GSE17538 cohort, and we used the same formula to calculate the risk score of the patients in this cohort. Similarly, patients were divided into high- and low-risk groups (**Figure 3G**). Patients with higher risk scores had poor DFS in the GEO cohort (P<0.05), which is consistent with the previous analysis of the TCGA cohort (**Figure 3H**).

Furthermore, we compared the risk score in the clinical characteristics of the TCGA cohort (age, cancer type, sex, stage and cancer site), and the results showed that the risk score was significantly different in stages and cancer sites (*P*< 0.05) (**Supplementary Figure 4A–E**). As the stage increases, the risk score has an upward trend, and the risk score of left colon cancer is higher than that of right colon cancer.

### 3.5 The molecular and mutation characteristics of different m6A-GPI groups

We compared some potential factors that determine tumor immunogenicity in two subgroups, and the results indicated that the risk score was positively correlated with HRD, CNA, and mRNAsi (**Supplementary Figure 5**). HRD mainly include loss of heterozygosity (LOH), telomere allele imbalance (TAI), and large-scale transition (LST). These three indicators can be used to determine the genomic instability score (GIS) and then evaluate the HRD status. CNA, LOH, TAI, and LST represent the level of chromosome instability (24). The mRNAsi is an index that can assess the similarity between tumor cells and stem cells and is related to the active biological processes in stem cells and the high degree of tumor dedifferentiation (25).

**Figure 4:**
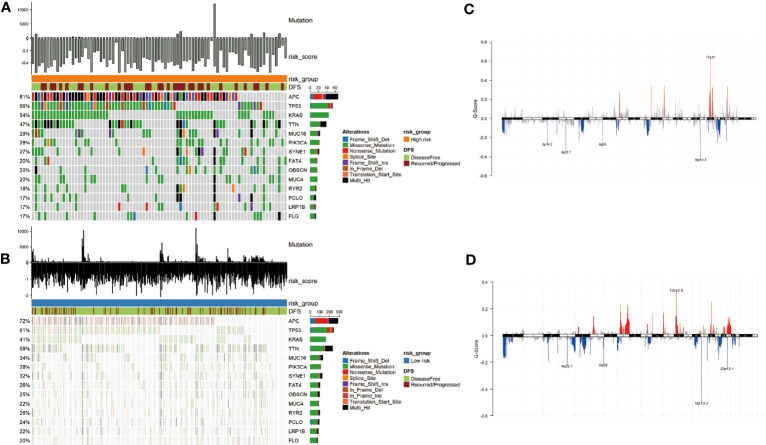
Mutational landscape and CNV of two groups in the TCGA-COAD. **(A, B)** Genes with high frequency mutation in the COAD samples of high-risk subgroup **(A)** and low-risk subgroup **(B)**. **(C, D)** CNV of the high- and low-risk groups. The markedly amplified part is displayed above the x-axis and marked with red; the markedly deleted part is displayed below the x-axis and marked with blue.

In addition, we used the “maftools” R package to analyze the distribution of somatic mutations between two subgroups in the TCGA cohort. Then we sorted the genes according to the mutation rate and identified the genes with the highest mutation rate in two groups. The mutation rates of APC, TP53, KRAS, TTN, MUC16, PIK3CA, SYNE1, FAT4, OBSCN, and MUC4 were higher than 20% in both groups (**Figures 4A, B**). After we grouped samples according to the risk score, the high-risk samples showed significant amplifications on chromosomes 8, 11, 12, 17, and 20, while deletions were found on chromosomes 1, 3 to 6, 8, 10, 12, 16 to 20. However, the low-risk samples showed significant amplifications on chromosomes 5, 6, 8, 10, 12, 13, 16, 17, 19, and 20, while deletions were found on chromosomes 1, 3 to 8, 10, 15 to 22 (**Figures 4C, D**).

### 3.6 Immune characteristics of different m6A-GPI groups

**Figure 5:**
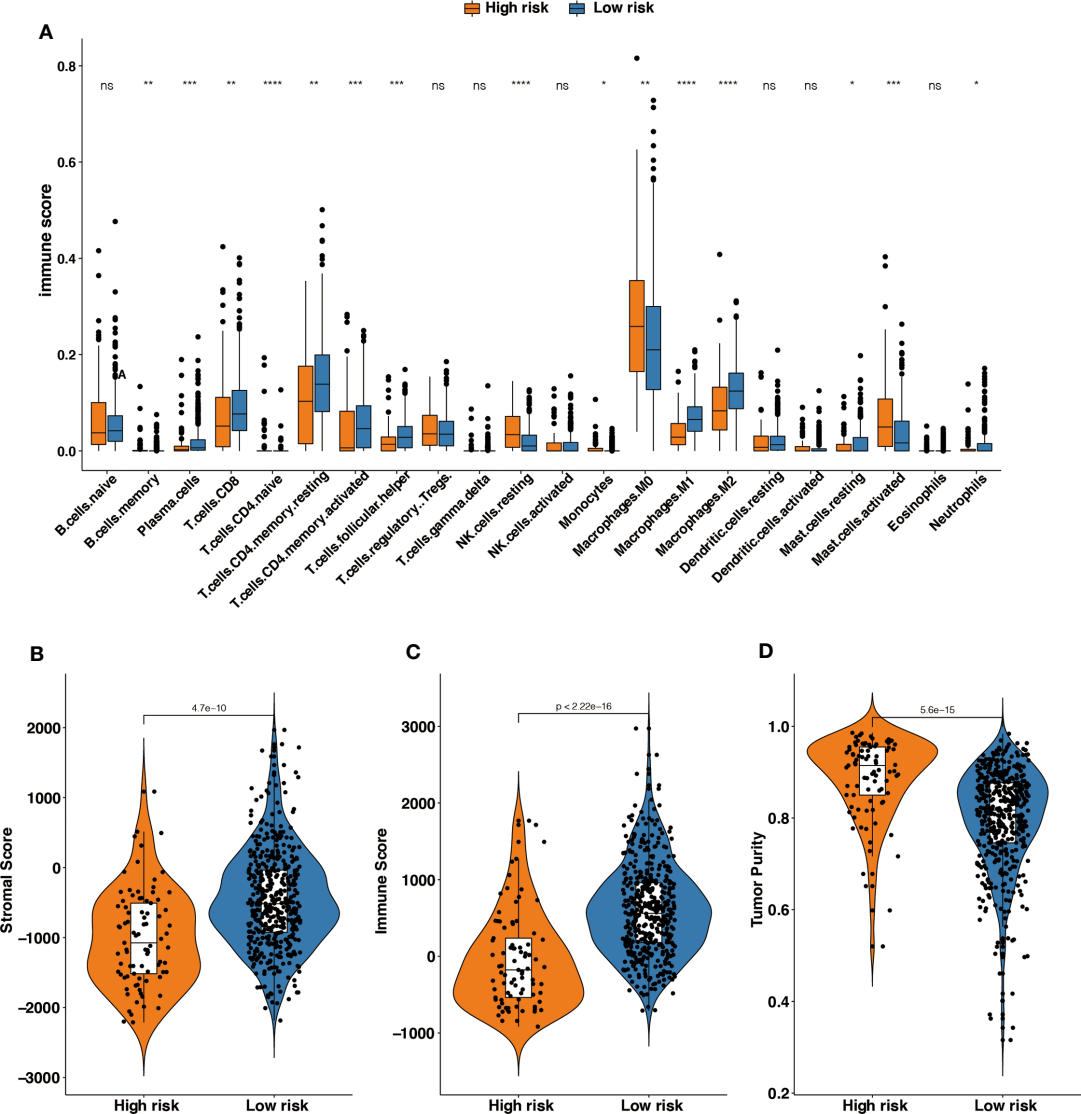
The different tumor-infiltrating immune cells between high- and low-risk groups based on m6A-GPI. **(A)** Profiles of 22 types of tumor-infiltrating immune cells in two groups. **(B-D)** Patients with a different stromal score, immune score, and tumor purity had different levels of risk scores. *p < 0.05; **p < 0.01; ***p < 0.001; ****p < 0.0001; ns, no significance.

The effect of tumor treatment depends not only on the tumor immunogenicity of the tumor but also on the TIME. TIME is formed by various cells, including immune cells (such as T cells, Tregs, NK cells, and macrophages), endothelial cells, and inflammatory mediators (26). The role of immune cells is particularly important, and it may affect the patient's response to treatment. To compare the distribution of immune cells in m6A-GPI subgroups, we analyzed the relative proportions of immune cells between the two m6A-GPI subgroups. Compared with low-risk patients, high-risk patients showed more infiltration of NK cells, M0 macrophages, and mast cells. However, in this group, there were fewer CD4+ T cells, CD8+ T cells, M1 macrophages, and M2 macrophages (**Figure 5A**).

Then we explored the level of the stromal score, immune score and tumor purity among the two groups, and the results showed that the high-risk group had higher tumor purity and that the low-risk group had a higher stromal score and immune score (**Figures 5B–D**). It suggests that the high-risk patients had a higher proportion of cancer cells in the tissue, and the TIME of the low-risk group contains contained abundant immune or matrix components.

### 3.7 Independent prognostic factor and nomogram

**Figure 6:**
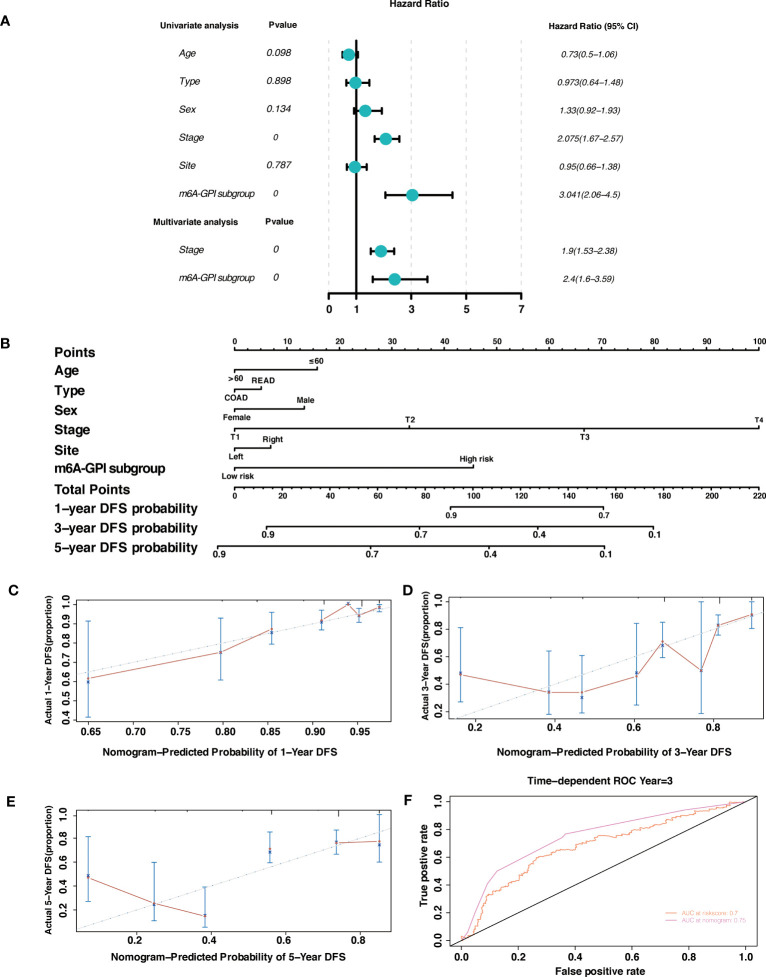
Independent prognostic factor and nomogram. **(A)** Univariate and multivariate analysis revealed that risk score based on m6A-GPI was an independent prognostic predictor. **(B)** Nomogram based on age, type, sex, stage, site, and risk group. **(C-E)** Calibration plots of the nomogram for predicting the probability of OS at 1-, 3-, and 5-year in the TCGA dataset. **(F)** The ROC curves of risk score and nomogram.

By using univariate Cox regression analysis and multivariate Cox regression analysis, we sought to determine whether m6A-GPI was an independent prognostic factor for patients with CRC. Univariate Cox analysis showed that m6A-GPI was closely related to the prognosis of CRC [hazard ratio (HR) = 3.041, 90% CI: 2.06–4.5, *P*< 0.001]. Multivariate Cox analysis further showed that m6A-GPI can be used as an independent predictor in CRC [hazard ratio (HR) = 2.4, 90% CI: 1.6–3.59, *P*< 0.001] (**Figure 6A**). At the same time, we constructed a nomogram and calibration plots of DFS at 1-, 3-, and 5-year in the TCGA dataset (**Figures 6B–E**), which provides doctors with a method to quantitatively predict the prognosis of CRC. The accuracy of prediction at 3 years can be increased to 0.75 after combining the risk score and clinical characteristics (**Figure 6F**).

### 3.8 Validation of m6A-related genes expression levels in CRC tissues

**Figure 7:**
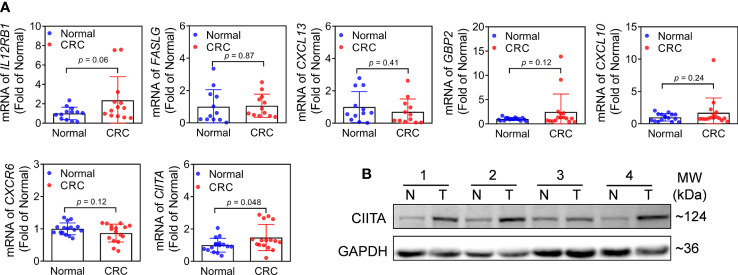
Expression of m6A-related genes in CRC and adjacent normal tissues. **(A)** mRNA expression levels of *IL12RB1*, *FASLG*, *CXCL13*, *GBP2*, *CXCL10*, *CXCR6*, and *CIITA* in CRC and adjacent normal tissues. n = 16, *p< 0.05 vs adjacent normal tissues. **(B)** Protein level of CIITA. Representative images were shown. n = 7, T: CRC tissues, N: adjacent normal tissues. Full-length blots of immunoblotting were presented in **Supplementary Figure 6**.

To further investigate the expression levels of m6A-related genes in CRC clinical tissues. We first detected the mRNA expression of m6A-related genes (*IL12RB1*, *FASLG*, *CXCL13*, *GBP2*, *CXCL10*, *CXCR6*, and *CIITA*). Notably, the expression of *CIITA* was substantially increased in CRC tissues (**Figure 7A**). However, there was no significant difference of others genes between CRC and adjacent normal tissues (**Figure 7A**). Subsequently, protein level of CIITA was detected in CRC and adjacent normal tissues. Consistent with mRNA levels, the protein level of CIITA was also obviously increased in CRC tissues (**Figure 7B**).

## 4 Discussion

Several lines of evidence indicate that m6A modification has become an important target in tumor immunity (27, 28). We believe that a novel prognostic marker based on m6A-related genes will help predict the prognosis and immune infiltration of colorectal cancer. For this reason, we screened 7 m6A-related genes (IL12RB1, FASLG, CXCL13, GBP2, CXCL10, CXCR6, and CIITA) through WGCNA and LASSO regression in this study and established an m6A-GPI in colorectal cancer. Survival analysis based on m6A-GPI showed that a lower risk score means longer DFS for patients. At the same time, we found that groups with different clinical characteristics (such as tumor site and stage) also showed differences in risk scores. We explored the molecular factors that affect tumor immunogenicity. The risk score was positively correlated with the HRD, CNA, and mRNAsi, and people with higher risk scores may have chromosomal instability. It is worth noting that m6A-GPI not only has good prognostic predictive ability but is also related to tumor immunogenicity, immune cell infiltration in CRC patients, which indicates that m6A-GPI may become a predictive indicator of tumor treatment. We also confirmed that m6A-GPI is an independent prognostic factor for CRC patients, which will provide useful guidance for clinical treatment strategies. Finally, we verified the expression levels of m6A-related genes (IL12RB1, FASLG, CXCL13, GBP2, CXCL10, CXCR6, and CIITA) in CRC tissues. Our results indicated that CIITA might play a crucial role in the prognosis of colorectal cancer.

At present, many studies have shown that m6A-related genes play an indispensable role in cancer progression and metastasis (29). Abnormal expression of genes associated with m6A has a significant impact on the prognosis of colorectal cancer. Overexpression of METTL3, a m6A writer, facilitates tumorigenesis of CRC by regulating the expression of genes related to cell cycle, noncoding RNA metabolism and glycolysis pathway (<xr rid="r36">^30^</xr>; 31, 32). The dysregulation of long non-coding RNA XIST, mediated by the loss of METTL14, has been found to be significantly associated with an unfavorable prognosis in patients with CRC (33). The m6A reader, YTHDC2, has been found to facilitate the metastasis of CRC by stimulating the translation of HIF-1α (34). However, the mechanisms by which m6A-related genes regulate immune cell infiltration in CRC remain elusive. In our study, the tumor immune microenvironment of patients with higher risk scores had increased infiltration of immune cells, such as resting NK cells, and M0 macrophages. Previous studies have shown that macrophages can be recruited to tumor tissues and contribute to tumor angiogenesis (35), which may cause poor DFS in high-risk groups. In addition, a significantly higher proportion of CD4^+^ T cells, CD8^+^ T cells, memory B cells, and M1 macrophages were found in patients in the low-risk group, indicating that there is a greater proportion of T cells and B cells in low-risk CRC tumors. CD8^+^ T cells play a major role in tumor immunity. CD8^+^ T cells differentiate into cytotoxic T cells in the body, and cytotoxic T cells can enter the tumor microenvironment and inhibit the growth of the tumor (36). In the TIME, there is a tendency for CD8^+^ T cells to increase in METTL3- or METTL14-null tumors, accompanied by increased secretion of IFN-γ, CXCL9 and CXCL10 (18). Meanwhile, studies have confirmed that the expression of ALKBH5 is specifically upregulated when T cells are activated, and ALKBH5 increases m6A modification on IFN-γ and CXCL2 in CD4^+^ T cells, thereby affecting mRNA stability and protein expression. These modifications lead to changes in the response of CD4^+^ T cells (37). The infiltration of M1 macrophages can promote inflammation and inhibit tumor cells in the TIME, M1 cells can be activated by IFN-γ and destroy tumors by producing nitric oxide, type 1 cytokines, and chemokines (38), which is consistent with the trend we observed in the low-risk group. Finally, this study found that compared with the high-risk group, CRC patients in the low-risk group had higher immune scores and stromal scores, and had lower tumor purity.

m6A modification changes the TIME, which largely affects the therapeutic response to antitumor immunotherapy. Approximately 85% of CRC patients have mismatch‐repair‐proficient or microsatellite instability‐low (pMMR-MSI-L) tumors. This type of patient failed to benefit from any single immunotherapy, but microsatellite instability-high (pMMR-MSI-H) CRC responds well to immunotherapy because it can recruit a large number of immune cells such as CD8^+^/CD4^+^ T cells and macrophages into the microenvironment (39–42). Wang et al. proposed that the destruction of m6A methyltransferase enhances the immunotherapy response of pMMR-MSI-L colorectal cancer by regulating the tumor microenvironment and tumor-infiltrating cells (18). In fact, the loss of METTL3 or METTL14 enhances the interaction between the tumor and the immune system through the IFN-γ-STAT1-IRF1. In another study on “eraser” ALKBH5, researchers found that the knockout of ALKBH5 in mice with CT26 colorectal cancer or B16 melanoma significantly reduced tumor growth and prolonged the survival rate of mice during immunotherapy. This may be related to ALKBH5 inhibiting immune cells in the tumor microenvironment and regulating lactic acid. These processes increase the response to anti-PD-L1 therapy and the loss of ALKBH5 changes the composition of immune cells and metabolite tumor microenvironment (13). In the subgroups based on m6A-GPI, the TIME and immune cells have changed. We found that tumors in the low-risk group recruited more CD4^+^/CD8^+^ T cells, our research provides important guidance for predicting the proportion of immune cells in CRC.

Our research provides ideal predictors for the prognosis and immune cell infiltration, but it is undeniable that there are several limitations in this study. First, we used retrospective data from public databases to construct and verify the m6A-GPI, and it would be more rigorous to use a larger-scale prospective data to evaluate its reliability. Second, the population in our study was mainly Americans, and different countries may have deviations in the results due to ethnic differences. In fact, this manuscript is the first part of our research, and our next research project will stem from these results.

In conclusion, m6A-GPI is a promising m6A-related prognostic biomarker. Our study divides patients into different risk subgroups based on m6A-GPI, which will help doctors identify the molecular and immune characteristics and predict the progression, prognosis of CRC. Moreover, m6A-GPI may be a potential indicator in the adjustment of tumor treatment strategies.

## Data availability statement

The datasets presented in this study can be found in online repositories. The names of the repository/repositories and accession number(s) can be found in the article/**Supplementary Material**.

## Ethics statement

The studies involving human participants were reviewed and approved by The Ethics Committee of the Liaoning Cancer Hospital, Shenyang, China. The patients/participants provided their written informed consent to participate in this study.

## Author contributions

BM conceived and designed this study. BM performed the bioinformatic analysis and visualization. SB performed the experimental validation. YL collected data and performed the statistical analysis. BM and SB wrote the original draft and BM revised the manuscripts. All authors revised and approved the final manuscript. All authors contributed to the article and approved the submitted version.
