# Supplementary material for: Identification and validation of m6A-GPI signatures as a novel prognostic model for colorectal cancer
Source: Front Oncol. 2023 Jun 23;13:1145753. doi: 10.3389/fonc.2023.1145753 (PMC10328717; doi:10.3389/fonc.2023.1145753)
Supplement: Supplementary file 1 [file DataSheet_1.pdf]

Supplementary Material

Identification and Validation of m6A-GPI Signatures as a Novel prognostic Model for Colorectal Cancer

Bin Ma*, Simeng Bao, Yongmin Li

* Corresponding Author: Bin Ma, mabin0326cmu@163.com

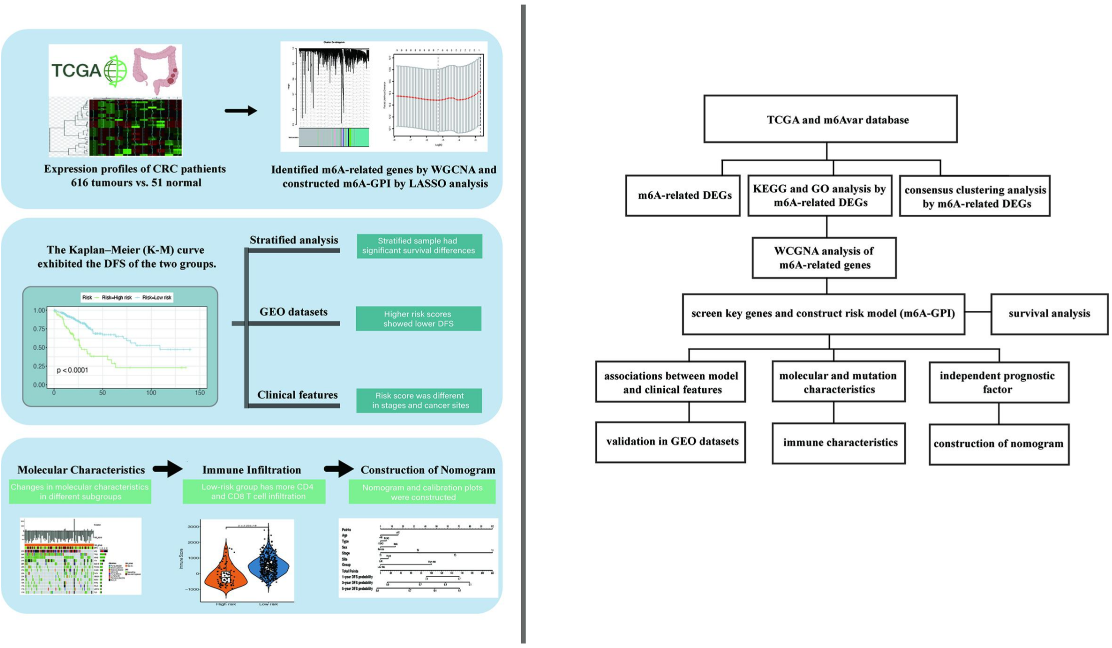

Supplementary Figure 1. The workflow of our study.

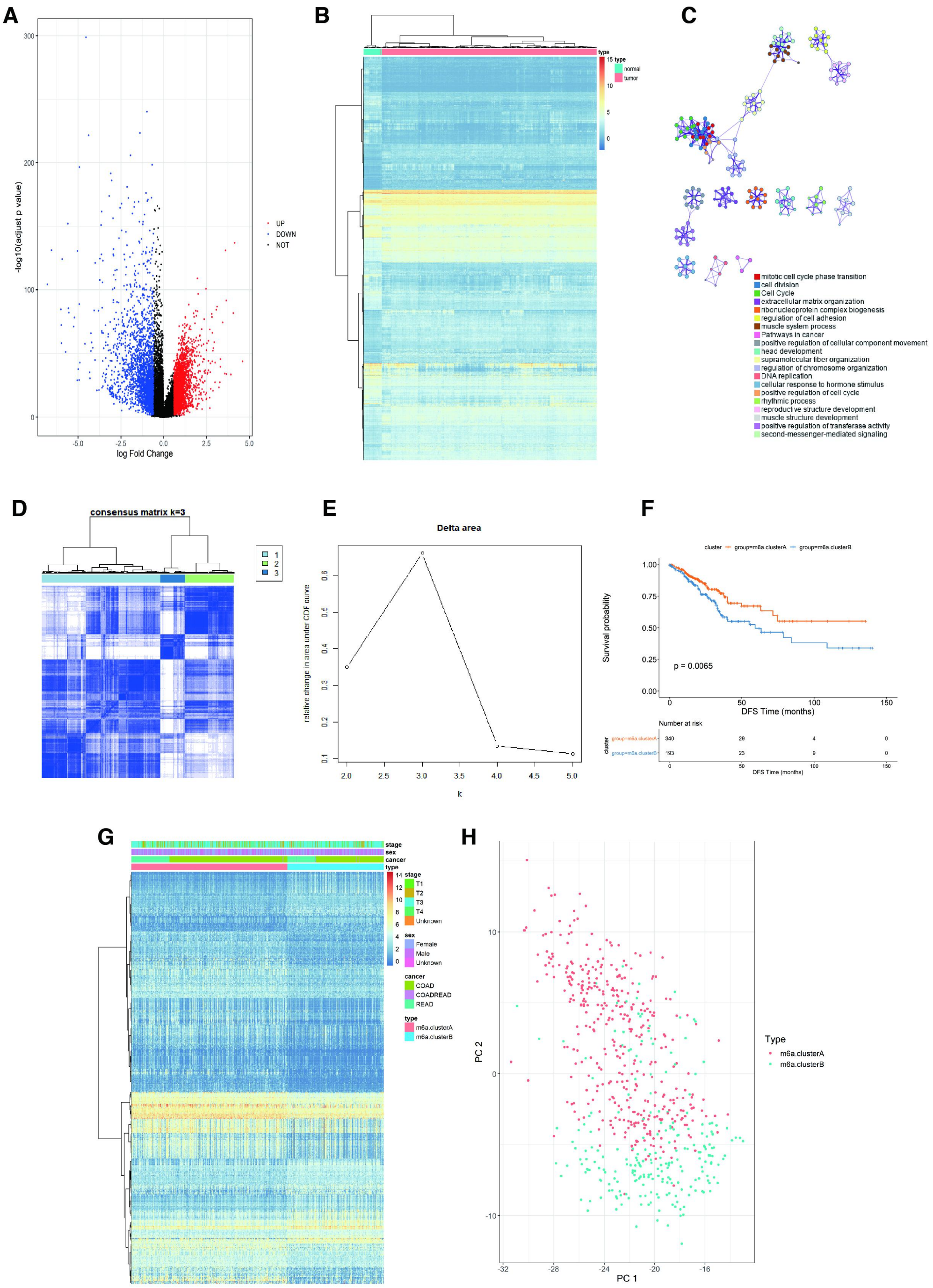

Supplementary Figure 2. The screening process of m6A-related genes.

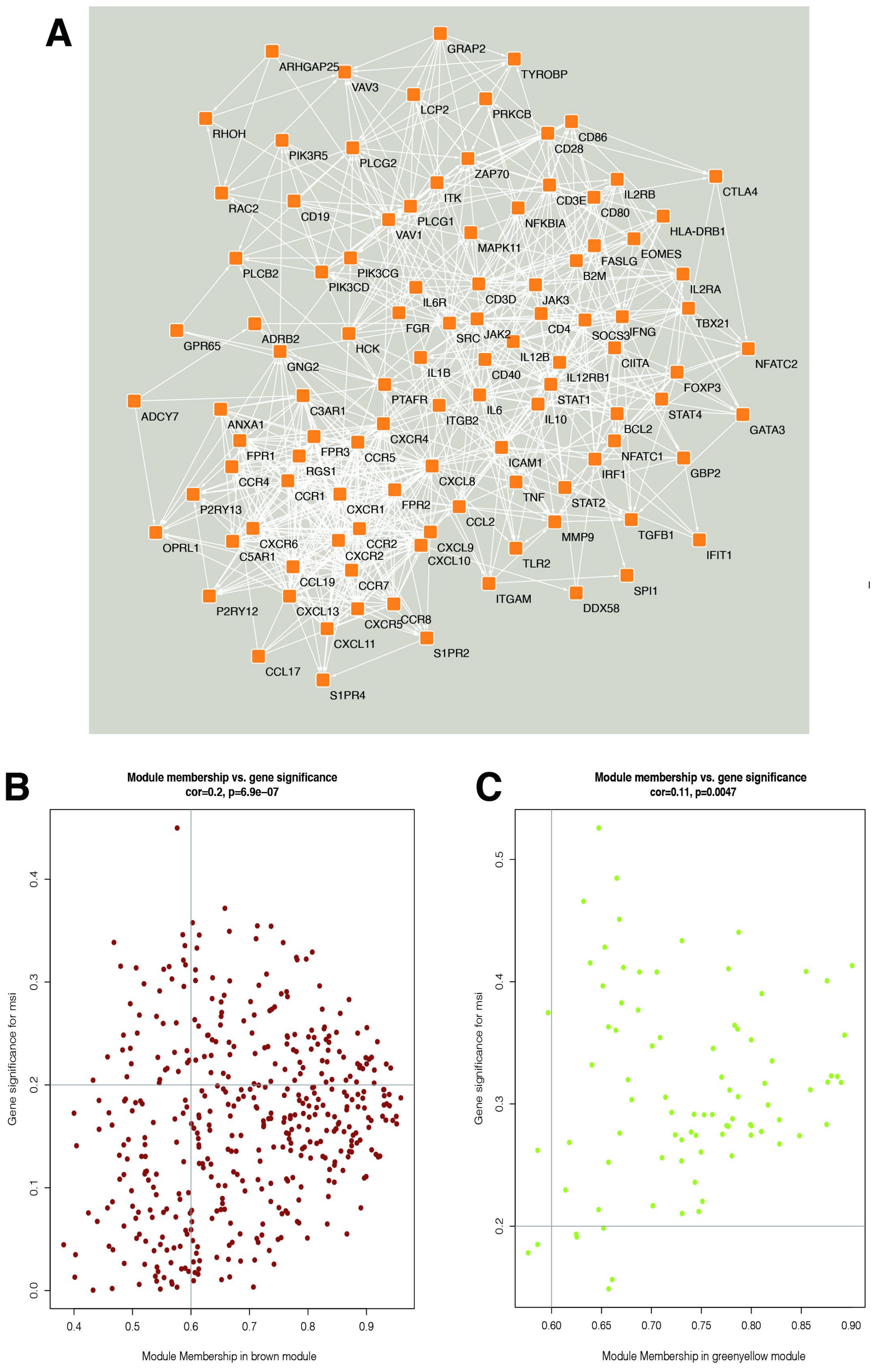

Supplementary Figure 3. (A) The protein-protein interaction (PPI) network based on two modules. **(B) (C)** Screening hub genes under the thresholds of module membership (MM) > 0.6 and gene significance (GS) > 0.2.

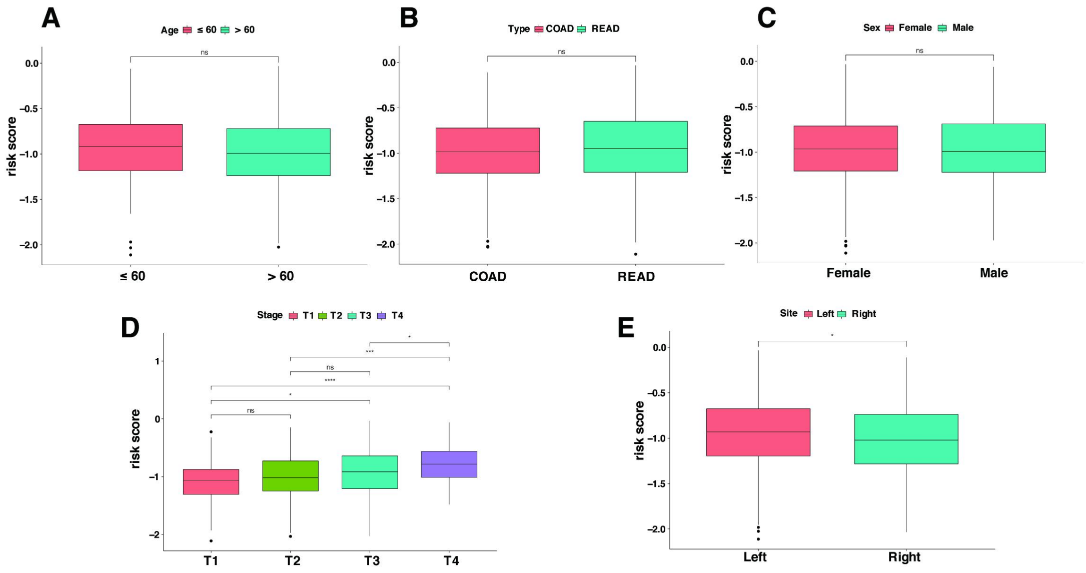

Supplementary Figure 4. (A-E) Patients with different clinicopathological features (including age, sex, type, stage, site) had different levels of risk scores, calculated based on the m6A-GPI.

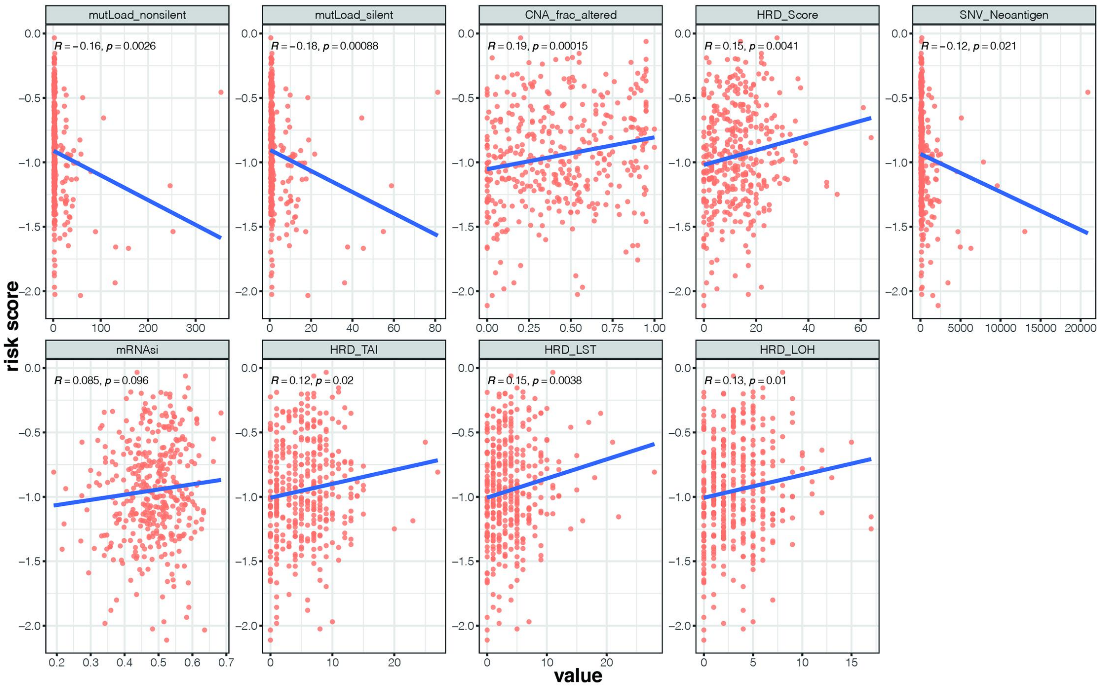

Supplementary Figure 5. Relationships between potential immune escape factors and the risk score based on m6A-GPI. The risk score was positively correlated with HRD, CNA, and mRNAsi.

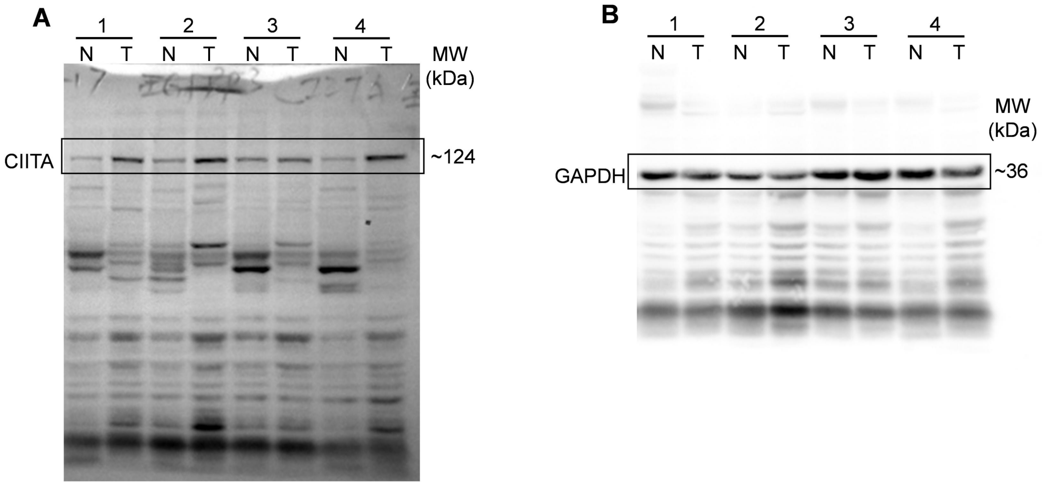

Supplementary Figure 6. Full-length blots of CIITA **(A)** and GAPDH **(B)**.
